# Structural Design of T-Cell Epitope-Based mRNA Vaccine Constructs Determines the Quality of T-Cell Immunity and Protective Efficacy Against SARS-CoV-2 in Mice

**DOI:** 10.3390/vaccines14030281

**Published:** 2026-03-23

**Authors:** Vladimir A. Gushchin, Andrei E. Siniavin, Andrei A. Pochtovyi, Alina S. Dzharullaeva, Dmitriy N. Shcherbinin, Anastasia S. Ungur, Amir I. Tukhvatulin, Inna V. Shuliakova, Denis A. Kleymenov, Elena P. Mazunina, Evgeniia N. Bykonia, Sofia R. Kozlova, Evgeny V. Usachev, Ilya D. Zorkov, Daria M. Grousova, Anna A. Iliukhina, Alexander L. Gintsburg, Denis Y. Logunov

**Affiliations:** 1 Federal State Budget Institution “National Research Center for Epidemiology and Microbiology Named After the Honorary Academician N.F. Gamaleya”, The Ministry of Health of the Russian Federation, 123098 Moscow, Russia; andreysi93@ya.ru (A.E.S.); alina-dzharullaeva@yandex.ru (A.S.D.); dim284@inbox.ru (D.N.S.); ungur.nastea@yandex.ru (A.S.U.); amir_tukhvatulin@gamaleya.org (A.I.T.); iv.dolzhikova@yandex.ru (I.V.S.); mne10000let@yandex.ru (D.A.K.); lenok27microb@gmail.com (E.P.M.); evgeniya_bikonya@mail.ru (E.N.B.); sofya_dadashyan@mail.ru (S.R.K.); evgenyvusachev@gmail.com (E.V.U.); a1460204@yandex.ru (I.D.Z.); grousova@gamaleya.org (D.M.G.); sovanya97@yandex.ru (A.A.I.); gintsburg@gamaleya.org (A.L.G.); ldy78@yandex.ru (D.Y.L.); 2 Department of Medical Genetics and Postgenomic Technologies, Sechenov First Moscow State Medical University, 119991 Moscow, Russia; 3 Department of Virology, Lomonosov Moscow State University, 119234 Moscow, Russia; 4 Department of Infectiology and Virology, Sechenov First Moscow State Medical University, 119991 Moscow, Russia

**Keywords:** mRNA vaccine, epitope-based vaccine, T-cell response, SARS-CoV-2, MHC I/II, CD8 T cells

## Abstract

Background/Objectives: Epitope-based mRNA vaccines represent a promising strategy for eliciting protective T-cell immunity against SARS-CoV-2 and as well as for non-infectious mRNA-based vaccines. However, how the structural architecture of vaccine constructs (including epitope arrangement, linker composition, signal peptide presence, and the combination of MHC class I and II epitopes) shapes the quality of T-cell responses remains poorly understood. Methods: Ten tandem minigene mRNA constructs (Cons1–10) encoding different combinations of MHC class I and class II epitopes from SARS-CoV-2 proteins (S, N, M, ORF3a) were designed, encapsulated in lipid nanoparticles, and administered to C57BL/6 mice. Immunogenicity was assessed by cytokine profiling (IFN-γ, IL-2, IL-4, IL-10) and T-cell proliferation assays. Protective efficacy was evaluated in K18-hACE2 transgenic mice challenged with SARS-CoV-2. Results: Constructs lacking a signal peptide and enriched in MHC class I-restricted epitopes induced robust Th1 responses and strong CD8^+^ T-cell proliferation, achieving up to 66% survival following lethal challenge. In contrast, constructs associated with elevated IL-10 and IL-4 production conferred limited protection (11–33%), consistent with functional skewing towards regulatory or Th2-associated immune profiles. Conclusions: These findings establish a direct link between construct design parameters and T-cell polarization quality, and provide a rational framework for next-generation epitope-based mRNA vaccine development.

## 1. Introduction

Since its emergence in December 2019, the novel coronavirus disease (COVID-19) has had a devastating impact on the world, resulting in more than 700 million cases and more than 7 million deaths worldwide [1,2]. The virus responsible for the COVID-19 pandemic, known as severe acute respiratory syndrome coronavirus 2 (SARS-CoV-2), has spread widely within the human population [3,4]. The virus genome is constantly undergoing mutations, leading to the emergence of new variants. Global vaccination programs have played a significant role in preventing the spread of SARS-CoV-2 infection [5,6,7,8,9].

A new mRNA-based platform for vaccine development and subsequent population immunization has become an effective approach to preventing SARS-CoV-2 infection and spread [10,11,12,13]. Nucleoside-modified LNP-mRNA vaccines are used worldwide and have significantly changed the course of the pandemic [14,15,16]. Well-known examples of such vaccines are BNT162b2 (Pfizer/BioNTech) and mRNA-1273 (Moderna), which used the whole S-glycoprotein of the SARS-CoV-2 virus as the antigen [17,18,19,20,21]. The S protein consists of two subunits: S1, responsible for binding to the ACE2 receptor, and the S2 subunit, which mediates the fusion of the virus with the host cell membrane. The S1 subunit contains a receptor-binding domain (RBD), which is the main target for the induction of neutralizing antibodies [17]. Most studies of SARS-CoV-2 vaccines have found a correlation between protection against infection and the presence of anti-S in the serum of vaccinated individuals [18,19]. Although the antigen composition of COVID-19 vaccines is regularly adapted to new virus variants, by the time they become widely available, a new subvariant of the virus will likely emerge, which will reduce the vaccine’s effectiveness [20,21]. Therefore, the development of next-generation vaccines that can provide protection against future SARS-CoV-2 variants by coordinating the antigen-specific immune cell responses induced by vaccination and/or infection is critical for the control of SARS-CoV-2.

While neutralizing antibodies provide the first line of defense by preventing viral infection of cells, a potent and sustained immune response relies heavily on cytotoxic CD8^+^ T lymphocytes, which are capable of eliminating infected cells by recognizing viral epitopes expressed on the cell surface by major histocompatibility complex class I (MHC-I) proteins [22,23,24,25,26,27]. Recognition by CD8 T cells of MHC-I-peptide complexes on the surface of antigen-presenting cells, such as dendritic cells, occurs through a specific T-cell receptor (TCR), leading to proliferation and differentiation of T cells into effectors that can kill the infected cell presenting antigen peptides by MHC-I [28,29]. Differentiation into antigen-specific memory CD8^+^ T cells (mCD8^+^), which can become effectors upon re-exposure, contributes to more durable control of the virus and the establishment of acquired resistance to infectious disease [27,30,31]. Effective control of viral infection further requires coordinated cooperation between CD4^+^ and CD8^+^ T cells. CD4^+^ T-helper (Th) cells are critical for maintaining cytolytic function and promoting CD8^+^ T-cell memory during infection [32,33,34,35]. In the context of SARS-CoV-2, antigen-specific CD8^+^ T cells have been shown to correlate with long-lasting immune protection and reduced disease severity [36,37,38,39]. Together, these observations highlight the importance of simultaneously engaging both CD4^+^ and CD8^+^ T-cell compartments through rational vaccine design—a principle that motivated the multi-epitope approach described in the present study [40,41,42]. An analysis conducted among patients after COVID-19 demonstrated the induction of T-cell responses against various viral proteins, including mainly the Spike (S) protein, nucleocapsid (N), membrane (M), ORF1 and ORF3 [43,44]. Using T-cell epitopes from these regions of the SARS-CoV-2 genome in immunization strategies could provide long-lasting and robust protection against emerging variants of the virus that evade the vaccine-induced humoral immune response [45,46,47,48]. Therefore, developing vaccines based on SARS-CoV-2 antigenic epitopes that effectively induce T-cell responses may be an effective vaccine strategy.

Multi-epitope constructs combining MHC class I and class II epitopes offer important advantages: MHC class II-restricted epitopes facilitate CD4^+^ T-cell help required for optimal CD8^+^ T-cell priming and memory formation [32,33,34,35]. Signal peptides directing antigens to the endoplasmic reticulum may promote MHC class I loading and cross-presentation [49,50]. Linker composition influences proteasomal processing efficiency. These design parameters have not previously been compared systematically in an LNP-mRNA platform, motivating the present study. Using the mRNA platform, we assessed the influence of the leader sequence, spacer composition, total peptide length containing MHC class I epitopes, and the presence of MHC class II epitopes on the specificity of T-cell responses to immunogenic regions in the S protein and the protective activity of tandem minigenes against SARS-CoV-2. Using a murine coronavirus infection model, we demonstrated that targeted optimization of these parameters significantly increases vaccine efficacy, protecting mice from death during viral infection. These findings provide a framework for the rational design of next-generation T-cell epitope-based vaccines.

## 2. Materials and Methods

### 2.1. Tandem Minigene Design

Protein sequence design was based on the IEDB database [51], where high-affinity T-cell epitopes from the Wuhan strain of SARS-CoV-2 were selected. For MHC class I epitopes (*H2-Kb* and *H2-Db* alleles), selection was performed from the IEDB database with subsequent affinity assessment using the MixMHCpred algorithm [52]. For MHC class II epitopes (*H2-IAb* allele), literature data [53] and the NetMHCIIpan 4.3e algorithm were used [54]. Next, epitopes were assigned a %Rank, representing the percentile rank of predicted binding affinity relative to a set of random natural peptides. The %Rank values were calculated with MixMHCpred for class I epitopes and with NetMHCIIpan for class II epitopes, respectively. Lower %Rank values indicate stronger predicted MHC binding (values > 2% were regarded as non-binders, 0.5–2% as weak binders, and ≤0.5% as strong binders). A total of 14 epitopes were selected: 3 for *H2-IAb*, 7 for *H2-Kb*, and 4 for *H2-Db*.

### 2.2. Animals Immunization and Challenge

All mouse experiments and procedures were approved by the Biomedical Ethics Committee of the Federal Research Centre of Epidemiology and Microbiology named after Honorary Academician N.F. Gamaleya (protocols #41 from 6 April 2023 and #56 from 31 July 2023). Female C57BL/6 mice (aged 5–6 weeks) were purchased from The Nursery of Laboratory Animals of the Institute of Bioorganic Chemistry of the Russian Academy of Sciences. All animals were housed under specific pathogen-free conditions in a temperature-controlled environment. The mRNA constructs encoding the full-length Spike from the Wuhan strain (LNP-mRNA-Spike) and T-cell epitopes (LNP-mRNA Cons1–10) were synthesized in vitro using a T7 RNA polymerase-mediated transcription reaction and encapsulated in a lipid nanoparticle as described previously [55,56,57]. C57BL/6 mice were intramuscularly injected with mRNA (10 μg per mouse) diluted in formulation buffer or formulation buffer alone (naïve). All mice were immunized with the studied LNP-mRNA three times with a 7-day interval between immunizations (*n* = 5 mice per group). The planned endpoint was 7 days after the third immunization, with euthanasia by CO_2_ inhalation followed by cervical dislocation.

The SARS-CoV-2 strain hCoV-19/Russia/Moscow_PMVL-1/2020 (lineage B.1.1.1, GISAID EPI_ISL_421275) was previously isolated from a nasopharyngeal swab from a patient with COVID-19 and propagated in Vero E6 cells (ATCC, CRL-1586). All experiments with SARS-CoV-2 were performed in biosafety level 3 (BSL-3) containment laboratories. For the protection studies, groups of vaccinated 9–10-week-old female hemizygous K18-ACE2-transgenic F1 mice (*n* = 9 per group) (males B6.Cg-Tg(K18-ACE2)2Prlmn/J (Jackson Laboratory, status SOPF) crossed with females C57BL/6Gamrc (Gamaleya Research Center, health status SPF)) were anesthetized with diethyl ether and intranasally challenged with 10^3^ TCID_50_ of SARS-CoV-2. Body weight and survival were monitored daily for 20 days to evaluate clinical indications of suffering and disease. C57BL/6 mice were selected for immunogenicity studies owing to their well-characterized H-2b MHC haplotype (*H2-Kb*, *H2-Db*, *H2-IAb*), which served as the direct basis for epitope selection. K18-hACE2-transgenic mice (sharing the C57BL/6 genetic background) were required for challenge experiments, as wild-type C57BL/6 mice are not susceptible to SARS-CoV-2 infection. The shared MHC haplotype minimizes haplotype-specific confounding in T-cell antigen recognition between the two models.

### 2.3. Isolation of Splenocytes

Spleens were collected on day 7 after the third immunization. For mechanical grinding, the spleen was smashed and passed through a 100 μm nylon cell strainer. Then, mononuclear cells (splenocytes) were isolated by Ficoll (PanEco, Moscow, Russia) density gradient centrifugation for 30 min at 700× *g*. Isolated cells were washed with phosphate-buffered saline (PBS; Sigma-Aldrich, St. Louis, MO, USA) and the number of viable cells was counted using a Countess 3 FL automatic cell counter (Thermo Fisher Scientific, Waltham, MA, USA).

### 2.4. Multiplex Cytokine Assay

A BioPlex Pro mouse cytokine 8-plex assay (Bio-Rad, Hercules, CA, USA) was used to detect cytokines in clarified splenocyte supernatants according to the manufacturer’s protocol. Mononuclear cells isolated from immunized mice were incubated with completed RPMI 1640 (HyClone, Logan, UT, USA) cell culture medium (supplemented with 10% fetal bovine serum (FBS; HyClone, Logan, UT, USA), 0.01 M HEPES, 0.05 mM mercaptoethanol (all from Thermo Fisher Scientific, Waltham, MA, USA), 2.98 mg/mL L-Glutamine, 2.5% sodium bicarbonate and penicillin (50,000 units)—streptomycin (50,000 µg) (all from PanEco, Moscow, Russia)). Next, the splenocytes (2 × 10^5^ cells in 200 µL/well) were incubated with the respective pool of peptides (5 µg/mL) or the full-length SARS-CoV-2 Spike protein (Sino Biological, Beijing, China) for 96 h at 37 °C. The peptides were synthesized by Synbio Tech Inc. (Taiwan, China; purity ˃ 98%). Phytohemagglutinin (PHA; 1 µg/mL; PanEco, Moscow, Russia) served as a positive control for T-cell responsiveness. Ovalbumin-derived peptides (OVA 257-264 and OVA 323-339; 5 µg/mL; Invitrogen by Thermo Fisher Scientific, Waltham, MA, USA) served as dual controls—to confirm in vitro restimulation capacity, and as antigen specificity controls—confirming the absence of cross-reactive responses in SARS-CoV-2-vaccinated animals. Unstimulated cells were included as a baseline negative control. The cell culture supernatant was collected and processed for measurement of cytokines. The results were analyzed by the MAGPIX^®^ Multiplexing System (Millipore, Burlington, MA, USA). The results were analyzed with GraphPad Prism version 10.1.

### 2.5. Proliferation Assay

Mouse splenic lymphocytes (5 × 10^6^ cells/mL) were resuspended in PBS and labeled with 0.5 μm CFSE (Thermo Fisher Scientific, Waltham, MA, USA) for 5 min at RT. Staining was terminated by washing the cells twice with PBS containing 1% FBS. After that, cells were plated into 96-well U-bottom plates (2 × 10^5^ cells in 200 µL/well) and stimulated with a pool of peptides (5 μg/mL for each peptide). Cells stimulated with PHA (1 µg/mL) were used as a positive control for non-specific T-cell stimulation. Cells stimulated with OVA 257–264 or OVA 323–339 peptides (5 µg/mL) were used as a control for non-specific stimulation with an irrelevant peptide, confirming the absence of cross-reactive proliferative responses. CFSE-labeled and unlabeled cells without peptide stimulation were used as negative controls. After being stimulated for 96 h, cells were harvested and washed with Stain Buffer (BD Biosciences, San Jose, CA, USA) and stained with anti-mouse CD3e-PE-Cy7, anti-mouse CD4-PE and anti-mouse CD8a-APC (Mouse T Lymphocyte Subset Antibody Cocktail; BD Pharmingen by BD Biosciences, San Jose, CA, USA). T-lymphocyte proliferation analysis was performed using a BD FACSAria III flow cytometer (BD Biosciences, San Diego, CA, USA).

## 3. Results

To systematically address how structural design parameters shape T-cell immunity, we formulated the following hypotheses for the present study: (1) inclusion of the HLA-B signal peptide would directly translate antigens to the endoplasmic reticulum, potentially enhancing antigen cross-presentation and MHC class I loading; (2) co-incorporation of MHC class II epitopes would engage CD4^+^ T-helper activity, thereby augmenting CD8^+^ T-cell priming; and (3) linker composition and epitope arrangement would modulate intracellular proteolytic processing efficiency, altering the balance between cytotoxic (Th1/CD8^+^) and regulatory T-cell polarization.

### 3.1. In Silico Analysis of Minigene Structure

The IEDB database was used for T-cell epitope selection. This tool is widely used for epitope prediction and analysis in humans, non-human primates, and other species. Seven H2-Kb epitopes, four H2-Db epitopes (a total of 10 epitopes, since one epitope binds to both alleles) and three H2-IAb epitopes with the highest %Rank values were selected (Table 1).

**Figure 1:**
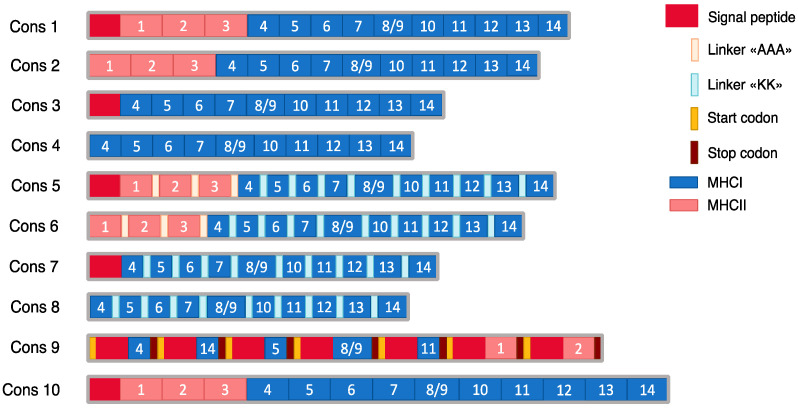
Schematic representation of constructs. The numbers indicate the location of the corresponding epitopes or peptides. The amino acid sequences are shown in Table 1.

Ten tandem minigene constructs (Cons) were designed based on the selected epitopes, each with distinct key features (Figure 1). For these constructs, both short epitopes (9–10 amino acids) and extended peptides (15–16 amino acids for MHC class I and 27 amino acids for MHC class II) were used, with each extended peptide containing the epitope centered within its native flanking sequences. Several constructs (Cons1, 2, 5, and 6) included three MHC class II epitopes (*H2-IAb*). Cons1, 3, 5, and 7 contained the HLA-B signal peptide, which can enhance antigen cross-presentation by targeting antigenic epitopes to the endoplasmic reticulum. MHC class II epitopes were fused using a «KK» linker, whereas MHC class I epitopes were connected via an «AAA» linker. In the head-to-tail (direct fusion) constructs (Cons1, 2, 3, and 4), epitopes were joined directly without any linker sequences. A 27-mer construct (Cons10) was also generated, in which all peptides were extended to 27 amino acids in length. In addition, a polycistronic mRNA construct (Cons9) was designed in which seven MHC class I epitopes were independently translated, each under the control of its own signal peptide, utilizing the ribosomal leaky scanning mechanism. In the designed mRNA, the first peptide is translated from an ATG codon located within a moderate Kozak context (GCCATGT) [58]; the second to the sixth ATG codons are in good Kozak context (GCCATGC); and the last peptide is positioned within an optimal Kozak context (ACCATGG). It is expected that through the leaky scanning mechanism, processed epitopes suitable for MHC loading will be generated.

**Figure 2:**
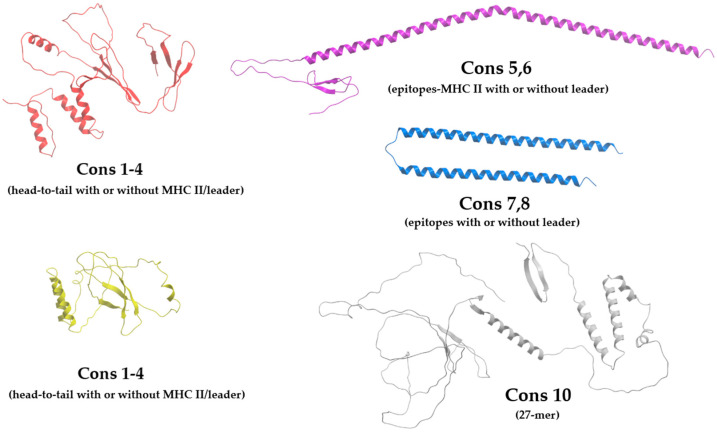
Structural modeling of tandem minigene constructs. Visualization of the tertiary structure was done by AlphaFold3 web service.

Structural modeling of the designed minigenes using AlphaFold3 indicated that the use of short linkers («AAA» or «KK») between epitopes favors the formation of α-helices structures, which could hypothetically contribute to epitope stability by reducing accessibility to exopeptidases (Figure 2). In contrast, the 27-mer construct was predicted to contain extensive intrinsically disordered regions, which may influence processing kinetics, though the in vivo consequences of this structural feature warrant further experimental investigation. However, such an interpretation would require biochemical validation, as AlphaFold3 does not model proteasomal processing or intracellular degradation kinetics.

### 3.2. Evaluation of the Influence of mRNA Structure on the Induction of T-Cell Response

**Figure 3:**
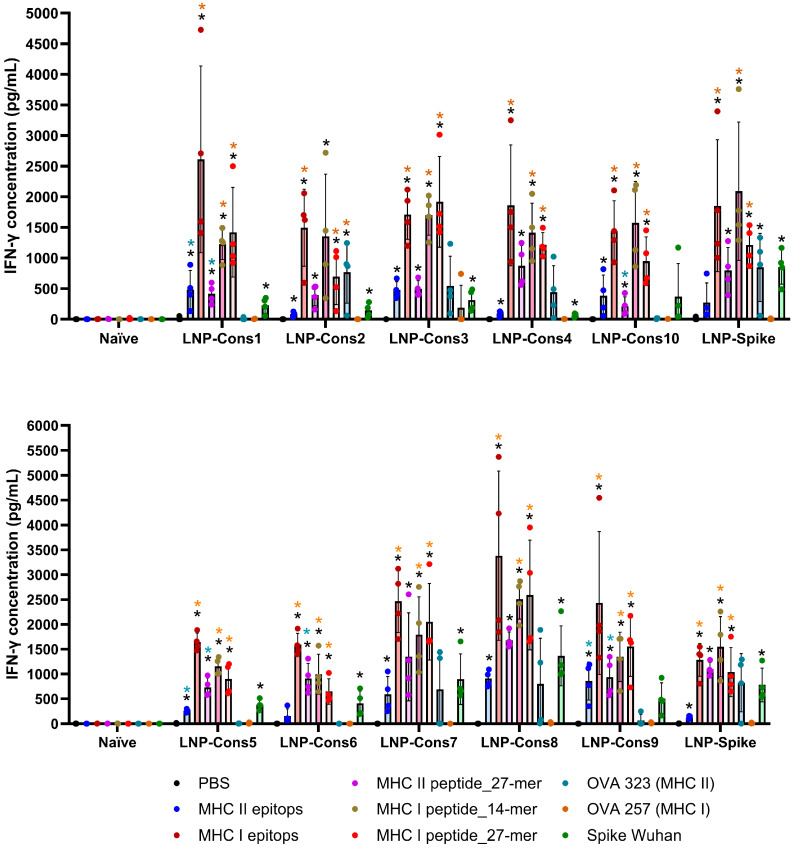
ELISA analysis of IFN-γ production in splenic mononuclear cells supernatants after restimulation, derived from mice vaccinated with different LNP-mRNA constructs. Error bars represent mean ± SD. Dots represent the values corresponding to each individual sample. According to legend, the color of the stars indicates the group to which they belong. Significance is indicated as * *p* < 0.05 and ** *p* < 0.01, tested by one-way ANOVA followed by Dunnett’s multiple-comparison test.

**Figure 4:**
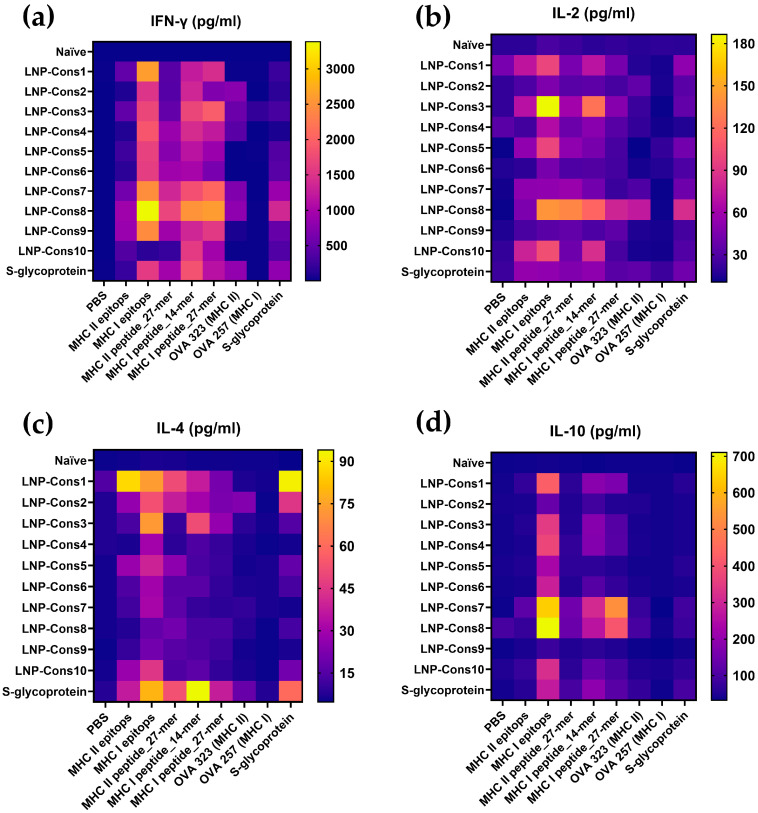
Heat map of immune mediators in the supernatant of splenic mononuclear cells collected from vaccinated mice. Cytokine levels of (**a**) IFN-γ, (**b**) IL-2, (**c**) IL-4 and (**d**) IL-10 measured by multiplex assay in the culture supernatants 72 h after restimulation.

We next studied the immunogenicity of ten mRNA constructs encoding peptides or epitopes of the SARS-CoV-2 virus. Seven days after vaccination with the third dose of lipid nanoparticle (LNP)-formulated mRNA constructs, splenic mononuclear cells from C57BL/6 mice were evaluated by restimulation with a mixture of MHC class I and MHC class II epitopes, 14- and 27-mer MHC class I and 27-mer MHC class II peptides, or the full-length SARS-CoV-2 Spike protein. Cells treated with PBS or ovalbumin MHC class I (SIINFEKL) and MHC class II (ISQAVHAAHAEINEAGR) peptides served as negative controls. Cell supernatant was collected 72 h after restimulation, and IFN-γ production was analyzed (Figure 3 and Figure 4a).

IFN-γ production by splenic mononuclear cells was significantly increased in all groups of animals immunized with LNP-Cons1 through LNP-Cons10 and LNP-Spike upon restimulation with MHC class I epitopes, as well as with 27-mer and 14-mer MHC class I peptides and the 27-mer MHC class II peptide. In response to MHC class II epitope restimulation, IFN-γ secretion was also significantly elevated in all immunized groups, except for LNP-Cons6. Restimulation of mononuclear cells with the full-length Spike protein resulted in a significant increase in IFN-γ levels in all groups except LNP-Cons9 and LNP-Cons10. No increase in IFN-γ production was observed in non-immunized (naïve) animals.

Importantly, restimulation with a non-specific ovalbumin peptide restricted to MHC class I (OVA_257) did not induce a significant IFN-γ response, whereas the MHC class II-restricted ovalbumin peptide (OVA_323) caused a moderate non-specific elevation of IFN-γ levels in cell supernatants obtained from LNP-Cons2-immunized animals. This non-specific response is most likely attributable to bystander T-cell activation triggered by MHC class II-presented peptides in an inflammatory context, or to residual LNP-mediated innate immune priming. Endotoxin contamination was excluded by manufacturer’s certificate quality.

The highest IFN-γ values were detected in mononuclear cells derived from mice immunized with LNP-Cons1, LNP-Cons7, and LNP-Cons8, although these differences were not statistically significant compared to the other groups. The strongest IFN-γ responses were observed upon restimulation with MHC class I epitopes and the 27-mer MHC class II peptide. Based on these findings, these antigens were selected for subsequent cellular restimulation experiments.

To gain further insight into the T-cell-mediated immune response induced by the LNP-mRNA constructs, the production of IL-2, IL-4 and IL-10 was also examined in splenic mononuclear cell culture supernatants following antigen restimulation (Figure 4b–d).

The highest IL-2 production was observed upon restimulation with peptides and epitopes selected for MHC class I, indicating a predominant cytotoxic T-cell-mediated immune response (Figure 4b). The greatest mean IL-2 concentrations were detected in the LNP-Cons1, LNP-Cons3, LNP-Cons5, LNP-Cons8, and LNP-Cons10 groups.

Increases in IL-4 concentration in the culture medium were most pronounced in the LNP-Cons1, LNP-Cons3, and LNP-Spike groups (Figure 4c), suggestive of functional skewing toward a Th2-associated response profile, though definitive lineage characterization would require intracellular cytokine staining (ICS) and transcription factor analysis (GATA3, T-bet). Notably, in the LNP-Cons3 and LNP-Spike groups, IL-4 production was also markedly enhanced following restimulation with the full-length Spike protein. The induction of a Th2-biased response in the LNP-Spike group was further supported by ELISA results, which showed a mean geometric antibody titer of 1:25,600 seven days after the final immunization. In contrast, no antibodies against the full-length SARS-CoV-2 Spike protein were detected in sera from animals immunized with the LNP-Cons1–10 constructs.

The production of the anti-inflammatory cytokine IL-10 in response to MHC class I-restricted peptides and epitopes was increased in all groups of immunized animals (Figure 4d). However, IL-10 upregulation following restimulation with MHC class II-restricted peptides was observed only in the LNP-Cons7 and LNP-Cons8 groups, which is suggestive of a partial tendency towards a regulatory immune profile; formal confirmation would require FoxP3 expression analysis.

**Figure 5:**
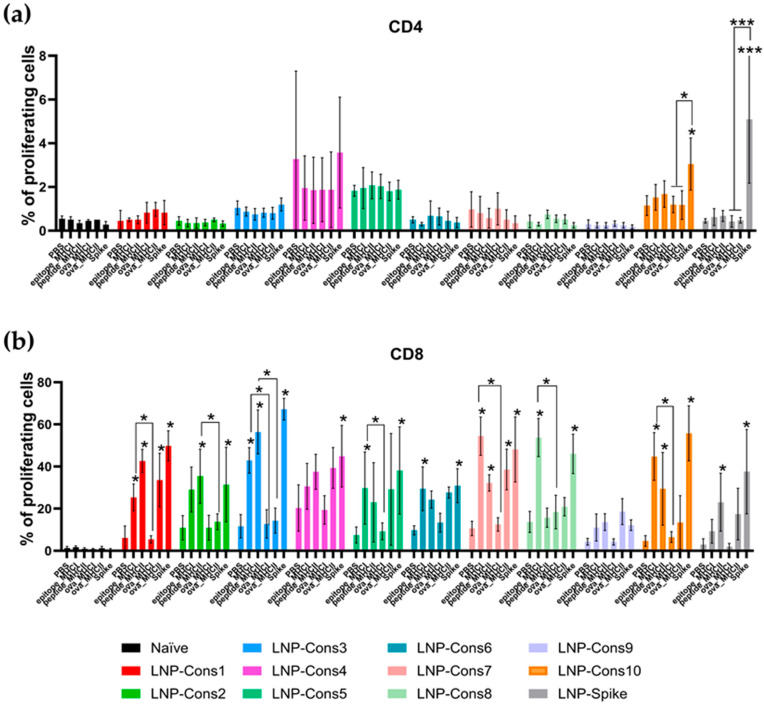
Antigen-specific T-cell proliferation. Splenocytes from immunized mice were stimulated with MHC class I epitopes or MHC class II peptides for 72 h. Proliferation of (**a**) CD4 and (**b**) CD8 T cells was detected using CFSE-based staining. Histograms show the mean ± SD for each sample group. Statistical significance was determined by two-way ANOVA followed by Dunnett’s multiple-comparison test. * *p* < 0.05, *** *p* < 0.001.

Since cytokine secretion represents only one aspect of T-cell activation, the next step was to assess the antigen-specific proliferative capacity of T cells. Proliferation was analyzed by flow cytometry 72 h after restimulation with the MHC class I-restricted epitopes, the 27-mer MHC class II peptide, the full-length Spike protein, and control ovalbumin-derived peptides. CD4^+^ and CD8^+^ T-cell proliferation was measured using the carboxyfluorescein succinimidyl ester (CFSE) dye (Figure 5).

A significant increase in the proportion of proliferating CD4^+^ T lymphocytes was observed only upon restimulation with the full-length Spike protein in the LNP-Cons10 and LNP-Spike groups (Figure 5a). Restimulation with the MHC class I-restricted epitope resulted in a significant increase in CD8^+^ T-cell proliferation in the LNP-Cons1, LNP-Cons3, LNP-Cons5, LNP-Cons6, LNP-Cons7, LNP-Cons8, and LNP-Cons10 groups compared to the PBS-treated control group (Figure 5b). Similarly, stimulation with the 27-mer MHC class II-restricted peptide resulted in a significant enhancement of antigen-specific CD8^+^ T-cell proliferation in the LNP-Cons1, LNP-Cons2, LNP-Cons3, LNP-Cons7, and LNP-Cons10 groups. Restimulation with the full-length Spike protein significantly increased antigen-specific CD8^+^ T-cell proliferation in all groups except LNP-Cons9. It should be noted that restimulation with the control MHC class II-restricted ovalbumin peptide (ova_MHC II), but not with the MHC class I-restricted peptide (ova_MHC I), induced a non-specific increase in CD8^+^ T-cell proliferation in cells from animals immunized with LNP-Cons1 and LNP-Cons7.

Overall, all constructs tested were immunogenic but differed in the qualitative characteristics of the elicited immune response. Immunization with LNP-Cons7 and LNP-Cons8 was associated with the most pronounced anti-inflammatory (regulatory) profile, whereas LNP-Cons4 and LNP-Cons9 failed to induce significant antigen-specific CD8^+^ T-cell proliferation.

### 3.3. Protective Response in K18-hACE2 Transgenic Mice Vaccinated with SARS-CoV-2 LNP-mRNA

**Figure 6:**
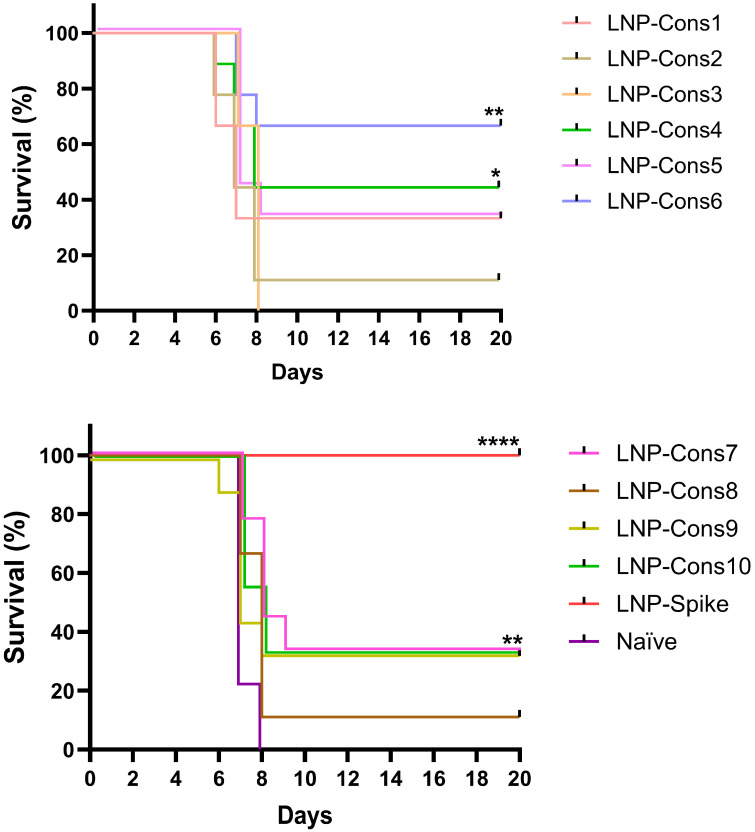
Survival of mRNA-vaccinated mice following challenge with SARS-CoV-2 virus. Mice (*n* = 9 per group) were vaccinated with the indicated mRNA constructs. Seven days following the final vaccination, mice were challenged with 10^3^ TCID_50_ SARS-CoV-2 (Wuhan-like strain). Survival was monitored for 20 days after the challenge. Asterisks indicate groups that were significantly different from the naïve (PBS) group: * *p* < 0.05, ** *p* < 0.01, **** *p* < 0.0001 as compared by logrank (Mantel–Cox) test.

Following the assessment of cellular immune responses, the next step was to evaluate the protective efficacy of the vaccine constructs using a lethal murine model of SARS-CoV-2 infection. Mice were immunized with LNP-mRNA constructs encoding different antigen designs, and survival rates were used to determine the level of protection (Figure 6). Protection experiments were performed with nine animals per group (*n* = 9), and survival percentages therefore correspond to 1–6 surviving animals depending on the construct.

Interestingly, the observed protection rates generally correlated with the patterns of T-cell responses described above. Constructs that elicited strong IFN-γ and IL-2 production, together with robust CD8^+^ T-cell proliferation such as LNP-Cons4, LNP-Cons6, and to a lesser extent LNP-Cons1, provided the highest levels of survival (44%, 66%, and 33%, respectively). These constructs were characterized by the presence of MHC class I-restricted epitopes and the absence of a signal peptide, which likely promoted efficient cytosolic antigen processing via the MHC class I pathway, leading to potent Th1-type and cytotoxic responses.

In contrast, constructs associated with elevated IL-4 or IL-10 levels (LNP-Cons7 and LNP-Cons8) induced a more regulatory or Th2-skewed cytokine profile, consistent with their lower protective efficacy (11–33%). The pronounced IL-10 production in these groups (Figure 4d) and the limited CD8^+^ T-cell proliferation (Figure 5b) suggest that excessive regulatory signaling may suppress effective cytotoxic immunity. Similarly, constructs containing extended peptides or the MHC class II epitopes (such as LNP-Cons2 and LNP-Cons3) generated mixed Th1/Th2 responses but were only partially protective, with mouse survival below 35%.

The polycistronic construct (LNP-Cons9) and the 27-mer construct (LNP-Cons10) demonstrated intermediate immunogenicity and protection (~33%), inducing detectable level of IFN-γ and IL-2 but relatively modest T-cell proliferation.

Body weight was monitored daily as a clinical indicator of disease progression during the challenge experiment. Animals reaching the predefined humane endpoint of 25% body weight loss were euthanized and counted as non-survivors. Consequently, weight-loss dynamics closely paralleled the survival curves shown in Figure 6. Viral load measurements were not performed in the present study because the primary aim of the challenge experiment was to compare survival outcomes across different vaccine construct architectures rather than to quantify viral clearance kinetics.

As expected, vaccination with the full-length Spike mRNA (LNP-Spike) provided complete protection (100%), reflecting a balanced induction of humoral and cellular immune responses, including Th1/Th2 cytokine production and robust antigen-specific proliferation. Although the multi-epitope constructs achieved lower absolute survival rates, the epitope-based approach retains several advantages that justify its further development. Constructs can be designed around conserved regions less susceptible to mutational escape across variants, potentially conferring broader cross-variant coverage [46,47]. T-cell immunity is particularly relevant in individuals with pre-existing Spike-specific antibody responses, where T-cell activity may compensate for humoral escape. Targeting non-Spike antigens (nucleocapsid, membrane protein, ORF3a) additionally broadens the antigenic target space. Furthermore, the absence of B-cell epitopes eliminates the theoretical risk of antibody-dependent enhancement. As expected, no anti-Spike antibodies were detected in any LNP-Cons1–10 group, confirming that the protective efficacy of these constructs is attributable entirely to T-cell-mediated mechanisms, directly supported by the observed correlation between CD8^+^ proliferation, IFN-γ/IL-2 levels, and survival. Overall, these results indicate that constructs favoring a Th1-dominant, cytotoxic CD8^+^ T-cell response confer the highest level of protection against SARS-CoV-2 infection, whereas those skewed toward Th2 or regulatory responses provide only partial or negligible protection. The findings underscore the critical role of antigen architecture, including linker composition, MHC targeting, and signal peptide presence, in determining the magnitude and quality of protective immunity elicited by mRNA vaccines.

## 4. Discussion

Recently, the design of vaccines based on cytotoxic T-lymphocyte epitopes has been actively used to develop peptide and mRNA vaccines, including for SARS-CoV-2 [59,60,61,62,63]. Epitope-based vaccines offer several advantages, including lack of autoreactivity, increased immunogenicity, and stability. The use of various immunoinformatic algorithms allows for the identification of effective epitopes for inclusion in vaccines [64,65,66].

In this study, we systematically evaluated ten different LNP-formulated mRNA constructs encoding varied epitope/peptide combinations derived from SARS-CoV-2, differing in MHC-I and MHC-II targeting, presence or absence of signal peptides, and tandem vs. “head-to-tail” (contiguous) design. The immunogenicity and protective efficacy were analyzed and compared.

Our cytokine data show that nearly all constructs induced elevated IFN-γ production upon restimulation with MHC class I epitopes, 14- and 27-mer MHC class I peptides, and the 27-mer MHC class II peptide. Specifically, constructs LNP-Cons1, LNP-Cons7 and LNP-Cons8 demonstrated the highest mean IFN-γ levels, although not statistically significantly superior to others. IL-2 secretion, indicative of T-cell activation and expansion, was highest in LNP-Cons1, LNP-Cons3, LNP-Cons5, LNP-Cons8 and LNP-Cons10, especially after MHC I-directed stimulation, supporting a cytotoxic T-cell-driven profile. Conversely, elevated IL-4 secretion (notably in LNP-Cons1, LNP-Cons3 and LNP-Spike) indicated a component of Th2 polarization alongside the Th1/cytotoxic response. IL-10 production, reflective of regulatory or anti-inflammatory signaling, was most marked in LNP-Cons7 and LNP-Cons8 after MHC I-targeting, and only in those groups after MHC II restimulation. Together with proliferation assays, which showed strong CD8^+^ T-cell proliferation in groups such as LNP-Cons1, LNP-Cons3, LNP-Cons5, LNP-Cons6, LNP-Cons7, LNP-Cons8 and LNP-Cons10, and only modest CD4^+^ proliferation (primarily in response to full-length Spike in LNP-Cons10 and LNP-Spike), these results present a nuanced picture: all constructs were immunogenic, but the quality of the response differed markedly.

When compared with survival outcomes in a mouse model of SARS-CoV-2 infection, there is a clear pattern of better protection associated with strong Th1/cytotoxic responses. Constructs such as LNP-Cons6 (66% survival) and LNP-Cons4 (44% survival), which induced elevated IFN-γ/IL-2 and robust CD8^+^ proliferation, demonstrated the highest protection. The observed correlation between T-cell functional profiles and protection is consistent with previously reported findings in coronavirus infection models. Several studies have demonstrated that the development of robust CD8^+^ T-cell responses, characterized by high IFN-γ production and cytotoxic activity, plays a critical role in viral clearance and survival following lethal challenge with SARS-CoV and SARS-CoV-2 [67,68,69]. In contrast, constructs with higher IL-10 or IL-4 dominance (LNP-Cons7, LNP-Cons8) or with weaker CD8^+^ proliferation (LNP-Cons9) provided only partial or minimal protection (11–33%). Immune responses dominated by IL-4 or IL-10 are often associated with insufficient viral control and delayed viral clearance, reflecting a shift toward Th2 or regulatory polarization that can limit effective cytotoxic responses [70,71]. The full-length Spike vaccine (LNP-Spike) achieved 100% survival, consistent with its combined induction of Th1/Th2 cytokines, CD4^+^ and CD8^+^ proliferation, and humoral responses. Although the multi-epitope constructs did not reach the level of protection observed with the full-length Spike vaccine in this homologous challenge model, epitope-based strategies offer several conceptual advantages. By focusing on conserved regions of multiple viral proteins (including nucleocapsid, membrane and ORF3a), such vaccines may provide broader cross-variant protection and reduce susceptibility to antigenic escape. Moreover, T-cell-focused vaccines may remain effective even when neutralizing antibody responses are reduced against emerging variants. Epitope-based designs can also improve population coverage by incorporating epitopes restricted by multiple MHC alleles and may serve as complementary boosters for individuals with pre-existing Spike-specific immunity.

As expected, anti-Spike antibody responses were not detected in animals immunized with any of the LNP-Cons1–10 constructs, consistent with the absence of B-cell epitopes in their design. ELISA analysis confirmed that only the LNP-Spike group developed detectable anti-Spike antibody responses, whereas sera from animals immunized with LNP-Cons1–10 constructs remained antibody-negative.

The protective function of these constructs is therefore attributable exclusively to T-cell-mediated immunity. This also confirms that the measured immune parameters reflect genuine T-cell-specific responses rather than contributions from humoral immunity.

Our findings align with and extend prior reports on T-cell-based vaccine strategies. For example, some mRNA vaccines against SARS-CoV-2 have been shown to induce broad CD4^+^ and CD8^+^ T-cell responses that correlate with reduced disease severity in humans [72]. Moreover, epitope-based mRNA vaccines encoding multiple conserved T-cell epitopes (rather than just the Spike protein) have been demonstrated to protect mice from lethal SARS-CoV-2 challenge, supporting the concept that T-cell immunity alone (in the absence of neutralizing antibodies) can confer protection [73]. In particular, the critical role of CD8^+^ T cells and IFN-γ production in antiviral protection has been demonstrated in other viral models where CD8^+^ T-cell-derived IFN-γ was sufficient to protect mice from infection even in the absence of CD4^+^ and B cells [74]. Our data show a comparable relationship: stronger CD8^+^ T-cell proliferation and IFN-γ induction showed a qualitative association with survival outcomes of mice following SARS-CoV-2 infection. Because challenge experiments were conducted under BSL-3 conditions, group sizes were necessarily limited (*n* = 9 per group), which restricts statistical power for detailed correlation analyses between immune parameters and survival.

Additionally, the shift toward regulatory or Th2-biased cytokines (IL-10/IL-4) has been associated in COVID-19 studies in humans with severe outcomes or immune dysregulation [75]. Thus, the relatively poorer protection seen with constructs promoting higher IL-10/IL-4 (LNP-Cons7, LNP-Cons8) is consistent with the literature suggesting that excessive regulatory signaling can dampen effective cytotoxic responses. Increased IL-4 levels suggest a tendency toward Th2-associated functional skewing; however, definitive lineage differentiation would require additional analyses such as intracellular cytokine staining and transcription factor profiling. While elevated IL-10 may reflect regulatory signaling, definitive identification of regulatory T-cell populations would require dedicated markers such as FoxP3 expression.

Although several constructs showed broadly comparable IFN-γ responses, protective efficacy varied. This likely reflects qualitative differences in cytokine balance and T-cell polarization rather than differences in mouse strain, as both immunogenicity and challenge experiments were performed in animals sharing the C57BL/6 genetic background.

The data highlight several design principles for epitope-based mRNA vaccines. Prioritizing MHC class I-restricted epitopes, optimizing for CD8^+^ T-cell activation, appears critical for protective efficacy. Minimizing signal peptides or design elements that shift processing toward MHC class II may reduce cytotoxic T-cell priming and thereby diminish protection in lethal challenge models. The inclusion or absence of the signal peptide also appeared to influence antigen processing and presentation efficiency.

Because SARS-CoV-2 infection occurs in the respiratory tract, lung-resident memory CD8^+^ T cells likely contribute substantially to protection. In the present study we focused on systemic T-cell responses using splenocytes in order to enable standardized comparison across multiple vaccine constructs. Future studies will include characterization of lung-resident immune populations to better define the local mechanisms of protection.

Constructs lacking the signal peptide tended to favor cytosolic translation and MHC class I-restricted presentation, correlating with stronger cytotoxic responses and improved protection. This observation is consistent with previous evidence that cytosolic antigen localization promotes effective CD8^+^ T-cell priming [49,50]. It should be acknowledged, however, that the relationship between signal peptide presence, ER targeting, and MHC class I presentation is more complex. In professional antigen-presenting cells—particularly dendritic cells—ER-directed antigens can undergo cross-presentation via TAP-dependent or vacuolar pathways, potentially enhancing rather than diminishing CD8^+^ T-cell priming. Furthermore, signal peptides may alter protein stability or secretion, and the LNP delivery context (muscle cell transfection versus APC uptake) further modulates antigen processing. Balanced cytokine responses (strong Th1 (IFN-γ, IL-2), moderate Th2, minimal regulatory IL-10) appear optimal. Epitope configuration (tandem vs. contiguous) and linker/signal peptide choices influence intracellular antigen localization and presentation, which in turn shape T-cell polarization.

## 5. Conclusions

In conclusion, our results demonstrate that the qualitative nature of T-cell responses, particularly CD8^+^ T-cell proliferation and IFN-γ/IL-2 secretion, is a strong correlate of vaccine-mediated protection in a lethal SARS-CoV-2 challenge mouse model. Constructs that skewed the immune profile towards regulatory or Th2 dominance were less protective, consistent with a suppressive role for IL-10 in cytotoxic immunity. A key limitation of the present study is the absence of lung immune profiling. Given the intranasal challenge route, characterization of lung-resident memory CD8^+^ T cells would be critical for understanding local protective mechanisms and translational relevance.

Received findings collectively underscore the importance of rational antigen design, encompassing epitope selection, intracellular targeting, and linker/signal peptide architecture for next-generation mRNA vaccines aimed at eliciting potent and durable cell-mediated immunity against SARS-CoV-2 as well as for non-infectious mRNA-based vaccines.

## Figures and Tables

**Table 1:** Sequences of epitopes and epitope-containing polypeptides with native flanking regions used for minigene design.

Allele	№	Epitope	Antigen	%Rank	Peptide	IEDB_ID
*IAb*	1	QRNFYEPQIITTDNT	S	0.1300	THWFVT**QRNFYEPQIITTDNT**FVSGNC	1310750
*IAb*	2	TDAVRDPQTLEILDI	S	0.7300	RDIADT**TDAVRDPQTLEILDI**TPCSFG	1313735
*IAb*	3	YFYYLGTGPEAGLPY	N	2.2300	DLSPRW**YFYYLGTGPEAGLPY**GANKDG	1313991
*Kb*	4	VVLSFELL	S	0.0104	YRV**VVLSFELL**HAP	1310002
*Kb*	5	RTLSYYKL	M	0.0186	ATS**RTLSYYKL**GAS	1330035
*Kb*	6	YNYLYRLF	S	0.0271	GGN**YNYLYRLF**RKS	1338972
*Kb*	7	AAYYVGYL	S	0.0826	AGA**AAYYVGYL**QPR	1309656
*Kb*	8	VLYENQKL	S	0.1328	TQN**VLYENQKL**IANQ	2243367
*Db*	9	VLYENQKLI	S	0.1719	TQN**VLYENQKLI**ANQ	1555419
*Kb*	10	FLPFFSNV	S	0.1586	QDL**FLPFFSNV**TWF	1329407
*Db*	11	DLLFNKVTL	S	0.1307	FIE**DLLFNKVTL**ADA	1392110
*Db*	12	LALLLLDRL	N	0.1791	DAA**LALLLLDRL**NQL	34851
*Db*	13	YLYALVYFL	ORF3a	0.7010	PFL**YLYALVYFL**QSI	1311604
*Kb*	14	VNFNFNGL	S	0.0010	NKC**VNFNFNGL**TGT	70066

## Data Availability

Data are contained within the article.
